# *Arabidopsis* redox status in response to caterpillar herbivory

**DOI:** 10.3389/fpls.2013.00113

**Published:** 2013-05-06

**Authors:** Jamuna Paudel, Tanya Copley, Alexandre Amirizian, Alberto Prado, Jacqueline C. Bede

**Affiliations:** Department of Plant Science, McGill UniversitySainte-Anne-de-Bellevue, QC, Canada

**Keywords:** *Arabidopsis thaliana*, caterpillar herbivory, cross-talk, induced defenses, signaling pathways, *Spodoptera exigua*

## Abstract

Plant responses to insect herbivory are regulated through complex, hormone-mediated interactions. Some caterpillar species have evolved strategies to manipulate this system by inducing specific pathways that suppress plant defense responses. Effectors in the labial saliva (LS) secretions of *Spodoptera exigua* caterpillars are believed to induce the salicylic acid (SA) pathway to interfere with the jasmonic acid (JA) defense pathway; however, the mechanism underlying this subversion is unknown. Since noctuid caterpillar LS contains enzymes that may affect cellular redox balance, this study investigated rapid changes in cellular redox metabolites within 45 min after herbivory. Caterpillar LS is involved in suppressing the increase in oxidative stress that was observed in plants fed upon by caterpillars with impaired LS secretions. To further understand the link between cellular redox balance and plant defense responses, marker genes of SA, JA and ethylene (ET) pathways were compared in wildtype, the glutathione-compromised *pad2-1* mutant and the *tga2/5/6* triple mutant plants. *AtPR1* and *AtPDF1.2* showed LS-dependent expression that was alleviated in the *pad2-1* and *tga2/5/6* triple mutants. In comparison, the ET-dependent genes *ERF1* expression showed LS-associated changes in both wildtype and *pad2-1* mutant plants and the ORA 59 marker *AtHEL* had increased expression in response to herbivory, but a LS-dependent difference was not noted. These data support the model that there are SA/NPR1-, glutathione-dependent and ET-, glutathione-independent mechanisms leading to LS-associated suppression of plant induced defenses.

## INTRODUCTION

As plants interact with multiple organisms, they need to prioritize their actions to respond appropriately. Plants manage this through synergistic or antagonistic interactions mediated through growth and defense hormones: a process known as cross-talk (Spoel and Dong, 2008; Robert-Seilaniantz et al., 2011). In plant–pathogen interactions, activation of the systemic acquired resistance (SAR) pathway by biotrophic pathogens may render the plant more susceptible to necrotrophic pathogens that elicit jasmonate (JA)- and ethylene (ET)-mediated responses (Glazebrook, 2005). Insect herbivores also exploit this plant hormone cross-talk to prevent the induction of defensive pathways (Felton and Korth, 2000); however, the mechanisms underlying this are not fully understood.

When tissues are damaged during caterpillar feeding, rapid changes in calcium signatures and the generation of reactive oxygen species (ROS), such as hydrogen peroxide (H_2_O_2_), leads to the induction of the JA pathway and plant defense responses (Lou and Baldwin, 2006; Arimura et al., 2011). At low, regulated concentrations, H_2_O_2_ is an important signaling molecule, however, uncontrolled levels are destructive as H_2_O_2_ readily reacts with cellular components (Schröder and Eaton, 2008; Forman et al., 2010). ROS is generated by mechanical damage but also by enzymes, such as glucose oxidase (GOX), present in the caterpillar’s labial saliva (LS; Eichenseer et al., 2010). In lima bean, the zone of H_2_O_2_ accumulation around the site of leaf damage is widened by ~500 μm by *Spodoptera littoralis* caterpillar herbivory compared to mechanical wounding (Maffei et al., 2006). This caterpillar LS-associated production of H_2_O_2_ is proposed to be a strategum of some insect species to interfere with induced plant defenses (Musser et al., 2002; Bede et al., 2006).

To avoid the detrimental effects of ROS, antioxidant proteins, such as superoxide dismutase, catalase, peroxidase, and the Halliwell–Asada (ascorbate/glutathione) cycle are activated to maintain cellular redox homeostasis (Noctor et al., 2012). The Halliwell–Asada cycle lowers cellular H_2_O_2_ levels through a series of redox reactions involving ascorbate and glutathione. Therefore, in response to stress, plants often alter the total glutathione pool or the ratio between oxidized to reduced glutathione (GSSG:GSH) to maintain low H_2_O_2_ levels. Recognition of biotrophic pathogen attack or salicylic acid (SA) mimic treatment may result in an increase in total glutathione levels (Fodor et al., 1997; Mou et al., 2003; Mateo et al., 2006; Mur et al., 2006). Infiltration of SA into *Arabidopsis* leaves initiates a transient oxidation of the glutathione pool 6 h after the time of injection (Mou et al., 2003; Mateo et al., 2006). In response to mechanical damage, the ratio of GSSG/total glutathione increases, reflecting an oxidized cellular environment, with oxidized glutathione (GSSG) positively linked to JA signaling (Mhamdi et al., 2010; Gfeller et al., 2011). *Arabidopsis* glutathione mutants are more susceptible to microorganism and insect attack (Ball et al., 2004; Parisy et al., 2007; Schlaeppi et al., 2008). *Arabidopsis* *pad2-1* mutant lacks γ-glutamylcysteine synthetase that catalyzes the first step in glutathione biosynthesis (Parisy et al., 2007); therefore, glutathione levels are approximately one-fifth wildtype levels. This line is more vulnerable to *S. littoralis* herbivory (Schlaeppi et al., 2008; Leon-Reyes et al., 2010; Mhamdi et al., 2010; Dubreuil-Mauriza et al., 2011). As well, as glutathione pools and ratio change, related processes, such as protein glutathionylation or *S*-nitrosylation that are also implicated in the regulation of defense against pathogens and herbivores, are affected (Wünsche et al., 2011; Espunya et al., 2012).

In response to caterpillar herbivory, the active form of JA, (+)-7-iso-jasmonoyl-L-isoleucine, bridges JA ZIM-domain (JAZ) proteins with the E3 ubiquitin ligase SCF^COI1^ complex, resulting in the proteasome-mediated degradation of JAZ and release of the basic helix-loop-helix transcription factor MYC2, responsible for the expression of JA-associated genes, such as *VSP2* and* LOX2* (Lorenzo et al., 2004; Dombrecht et al., 2007; Kazan and Manners, 2008; Fonseca et al., 2009; Robert-Seilaniantz et al., 2011). Caterpillar herbivory-related increases in ET biosynthesis may modulate these JA responses through cross-talk between the JA-dependent MYC2-branch and ET-dependent branches (Stotz et al., 2000; Bodenhausen and Reymond, 2007; Kazan and Manners, 2008; Diezel et al., 2009; Robert-Seilaniantz et al., 2011; Verhage et al., 2011). Two APETALA2/ERF transcription factors, ET response factor1 (ERF1) and ORA59 integrate ET cross-talk with the JA pathway (Penninckx et al., 1998; Lorenzo et al., 2003; Pré et al., 2008); though both these branches are induced by ET, evidence points to them being parallel and, perhaps, functionally redundant. Together, the MYC2 and ET pathways, ORA59/ERF1, act synergistically or antagonistically allowing the integration of temporal and spatial hormone concentrations and localization to generate a specific signal signature (Kazan and Manners, 2008; Robert-Seilaniantz et al., 2011).

Effectors in the caterpillar LS may also activate the SAR pathway leading to the attenuation of JA-dependent responses (Kazan and Manners, 2008; Weech et al., 2008; Leon-Reyes et al., 2010; Verhage et al., 2011). In recognition of attack by biotrophic pathogens, plants mount the systemic defense response, SAR, initiated by increases in cellular SA and H_2_O_2_ that positively impact each other’s production (Rao et al., 1997; Glazebrook, 2005; Mateo et al., 2006). The resultant change in glutathione redox balance results in the activation of the non-expressor of PR-genes1 (NPR1) through thioredoxin-catalyzed reduction of the disulfide bridges, changing the protein from its cytosolic oligomer form to the monomer that enters the nucleus (Spoel et al., 2009; Noctor et al., 2012). Association of NPR1 with TGA transcription factors leads to the expression of pathogenesis-related genes, such as *PR1*. The mechanistic basis of the antagonism between SA and JA pathways is still debated (Lorenzo and Solano, 2005). Early evidence suggests that SA interferes directly with JA biosynthesis (Doares et al., 1995; Rayapuram and Baldwin, 2007). However, NPR1 has been shown to be interfere with JA signaling downstream of JA biosynthesis (Mou et al., 2003; Spoel et al., 2003; Ndamukong et al., 2007; Koornneef and Pieterse, 2008; Spoel and Dong, 2008; Tada et al., 2008; Leon-Reyes et al., 2010). This may reflect the observation that ET modifies SA/NPR1 inhibition of JA responses such that in the presence of ET, the attenuation of JA-dependent gene expression is NPR1-independent; however, in the absence of ET, NPR1 is necessary to interfere with these responses (Leon-Reyes et al., 2009). Weech et al. (2008) used *Arabidopsis* mutants to show that caterpillar LS interference of JA-dependent plant defenses by activation of the SAR pathway requires an active NPR1. In addition, Diezel et al. (2009) showed that damage of wild tobacco by caterpillars of the tobacco hornworm, *Manduca sexta*, result in an ET burst that attenuates the SA-mediated suppression of plant defense responses. Therefore, in plant–caterpillar interactions, there appears to be extensive interplay between JA, SA, and ET pathways.

The present research is designed to understand the potential role of cellular redox balance in the ability of caterpillar LS to interfere with host plant defense responses. Since caterpillar LS contains redox enzymes, such as GOX that generate H_2_O_2_, caterpillar saliva should perturb the redox state or balance even more than mere wounding (Eichenseer et al., 2010; Noctor et al., 2012). By using normal caterpillars with intact LS secretions or insects where LS secretions have been impaired by cauterization of the spinneret, one can tease out the effect of LS on the modulation of plant responses. Therefore, in response to herbivory by caterpillars with intact or impaired LS secretions, the redox metabolites glutathione and ascorbate were measured to identify the impact of LS on cellular redox balance. As well, transcript responses of JA-, ET-, and SA-dependent marker genes were compared in wildtype plants and two mutant lines, *pad2-1*, compromised in glutathione biosynthesis, and a *tga2/5/6* triple mutant that is deficient in the basic leucine zipper TGA transcription factors that interact with NPR1 (Zhang et al., 2003; Parisy et al., 2007).

## MATERIALS AND METHODS

### PLANTS

*Arabidopsis* seeds ecotype Col-0 (TAIR CS3749) and the *pad2-1* mutant (TAIR CS3804) were obtained from the Arabidopsis Biological Resource Centre (Ohio State University). Seeds of the *Arabidopsis* *tga2/5/6* triple mutant were a generous gift from Dr. Li (University of British Columbia).

For redox metabolite experiments, wildtype plants seeds were surface-sterilized by soaking them for 2 min in 70% ethanol, followed by 5 min in 50% bleach. Seeds were rinsed three times in sterile distilled water and sown in Premier Promix BS (Premier Horticulture Inc.). After cold treatment at 4°C for 3 days, seeds were transferred into a growth cabinet (light intensity 140 μEm^-2^s^-1^, 12:12 light:dark at 22°C). Plants were bottom-watered as needed, about three times per week with dilute 0.15 g/L N–P–K fertilizer.

For gene expression experiments, seeds were surface-sterilized as described above and germinated on half-strength Murashige and Skoog (MS) media with 1% agar. After cold treatment for 3 days at 4°C, seeds were placed in the growth cabinet and transferred to Agro-Mix at germination. At 5 weeks post-germination, one plant from each genotype (Col-0, *pad2-1* and *tga2/5/6*) were transplanted into a 12.5 cm × 12 cm pot.

Approximately 6- to 7-week old plants in the late vegetative growth stage, between growth stages 3.7 and 3.9 according to Boyes et al. (2001), were used in redox metabolite or gene expression experiments.

### CATERPILLARS

Beet armyworm, *Spodoptera exigua* (Hübner; Lepidoptera: Noctuidae), insects were reared for multiple generations from eggs purchased from Bio-Serv (Frenchtown, NJ, USA). Insects were reared under defined conditions in a growth cabinet (16:8 light:dark, RH 28-40%, temperature 28.5°C) and fed a wheat germ-based artificial diet (Bio-Serv). Adult moths were allowed to mate and the eggs collected to maintain the colony.

### IMPAIRMENT OF CATERPILLAR LS SECRETIONS

Caterpillar LS is secreted through a specialized organ, the spinneret (Musser et al., 2002). To impair LS secretions, this spinneret was cauterized as previously described (Musser et al., 2002; Bede et al., 2006). Prior to the experiment, caterpillars were allowed to feed > 12 h on *Arabidopsis* plants to allow the insects to adjust to the plant diet.

### MEASUREMENT OF REDOX METABOLITES

Leaf H_2_O_2_ levels were not measured directly due to the high variability associated with the instable nature of this compound and confounding effects by high leaf phenolic content and ascorbate (Queval et al., 2008). Therefore, other metabolites associated with the ascorbate/glutathione cycle were measured since they closely correlate with H_2_O_2_ levels (Ng et al., 2007). Six-week-old *Arabidopsis* plants were subject to one of three treatments: untouched (control) or subject to herbivory by 3 × 4th instar *S. exigua* caterpillars with intact or impaired salivary secretions. As *S. exigua* caterpillars feed most actively at night, experiments were performed during the dark to more accurately simulate an ecological scenario. Rosette leaves showing signs of herbivory were harvested at 5, 15, 25, 35, and 45 min and immediately frozen in N_2_. The experiment was repeated thrice.****

At each time point, ascorbate and glutathione were measured in three to four independent samples. Plant samples were finely ground in liquid nitrogen and extracted in 0.2 N HCl at a ratio of 100 mg leaf/mL acid. This was followed by neutralization with NaOH as described in Queval and Noctor (2007). Chemicals used in redox metabolite assays were purchased from Sigma Chemical Company.

#### Ascorbate

Total, oxidized and reduced ascorbate from the leaf extract supernatant were determined by measuring reduced ascorbate levels spectrophotometrically at A_265_ using an Infinite M200 Pro microplate reader (Tecan) according to Queval and Noctor (2007). Total ascorbate was measured by converting dehydroascorbate (DHA) to the reduced form by incubating the supernatant in dithiothreitol (0.4% v/v) in 67.2 mM sodium phosphate (NaH_2_PO_4_) buffer, pH 7.5 for 30 min at room temperature. Triplicates of each sample were incubated with ascorbate oxidase (0.2 U) and reduced ascorbate was measured after an 8 min incubation. Reduced ascorbate (ASc) levels were measured by adding ascorbate oxidase to the neutralized leaf extract supernatant in 0.1 M sodium phosphate buffer, pH 5.6, incubating at room temperature for 30 min and analyzing as above. Concentrations were determined from a six-point L-ascorbate standard curve (40–240 μM). Oxidized ascorbate levels (DHA) were calculated by subtracting reduced from total ascorbate.

#### Glutathione

Measurement of glutathione is based on a recycling assay (Rahman et al., 2006; Queval and Noctor, 2007); glutathione reductase, in the presence of nicotinamide adenine dinucleotide phosphate (NADPH), catalyzes the reduction of GSSG to GSH that reacts with 5,5′-dithiobis(2-nitrobenzoic acid) (DTNB) forming 5-thio-2-nitrobenzoic acid (TNB) that can be measured spectrophotometrically at A_412_. Total glutathione was measured by incubating leaf supernatant in 0.6 mM DTNB and glutathione reductase (0.015 U) in 0.1 M sodium phosphate (NaH_2_PO_4_) buffer, pH 7.5. After the addition of 0.5 mM β-NADPH, the TNB chromophore was monitored at A_412_ at 5 s intervals for the first 2 min. Total glutathione concentration was calculated based on triplicate eight-point standard curve (100 nM to 60 μM). Oxidized glutathione (GSSG) was measured by removing any reduced GSH from the sample by precipitation with 2-vinylpyridine followed by conversion of GSSG to GSH and measurement using the glutathione reductase/β-NADPH/DTNB method as described above (Griffith, 1980; Rahman et al., 2006; Queval and Noctor, 2007). Briefly, leaf supernatant was incubated with 1 μl 2-vinylpyridine (approximately 10-fold above GSH levels) for 30 min at room temperature. After centrifugation at 13,000 rpm for 5 min to remove excess 2-vinylpyridine, samples were diluted in 0.1 M sodium phosphate buffer, pH 7.5 and assayed in triplicate. GSSG levels were determined from a triplicate eight-point GSSG standard curve ranging from 100 nM to 3.2 μM. Reduced GSH was calculated by subtracting 2 × GSSG from total glutathione.

### GENE EXPRESSION

Three days before the herbivory experiment, clear plastic bottles were placed around the plants with mesh covering the tops. *Arabidopsis* plants were subject to one of three treatments: untouched (control) or subject to herbivory for 36 h by 6 × 4th instar *S. exigua* caterpillars with intact or impaired salivary secretions. The experiment was repeated twice; at each time point, two independent samples were taken for gene expression analysis (total *n* = 3–4).

#### RNA extraction, cDNA synthesis, and quantitative real-time polymerase chain reaction

Plants were finely ground in liquid nitrogen and total RNA was extracted using the RNeasy Mini Kit (Qiagen) following the manufacturer’s protocols. After DNase treatment (Wipeout, QuantiTect Reverse Transcription kit; Qiagen), the absence of genomic contamination was confirmed using 5′-ATG GGT CGT CAT CAG ATT CAG AGC AGA TAA-3′ and 5′-CAT ATA AGA GGT GTG TTA GAG ACA ATA ATA-3′ primers which span an intron (Weech et al., 2008). One microgram of RNA was converted to cDNA using a QuantiTect Reverse Transcription Kit following the manufacturer’s instructions.

Gene-specific primers were identified from the literature or designed using Primer3 (Table A1 in Appendix). Transcript expression was analyzed in duplicate using the Brilliant One-Step quantitative RT-PCR kit (Stratagene), according to the manufacturer’s protocol, in a Mx3000p thermocycler (Stratagene). Gene amplicon products were verified by sequencing. Each 96-well plate, contained a standard curve of the gene-of-interest, a non-template control and each sample in duplicate. Each reaction contained 1× SYBR green I, 0.375 nM ROX, 100 nM of the forward and reverse primer, mastermix that contained dNTPs, MgSO_4_ and *Taq* polymerase, and either water (non-template control), serial dilutions of PCR amplicon (standard curve) or 85 ng cDNA sample. Standard curves ensured an efficiency of between 90 and 110%. Thermocycler conditions are as follows: 95°C for 10 min; 40 cycles of denaturation at 95°C for 45 s, annealing for 1 min, and elongation at 70°C for 45 s. The annealing temperature was dependent on the primers used (Table A1 in Appendix). Dissociation curves were performed to ensure amplicon purity. Two technical plate replicates were performed.

From the standard curve, gene copy numbers were estimated and normalized against the constitutive reference gene *AtACT2* (At3g18780). *Arabidopsis* *AtACT2* expression was not affected by osmotic stress or when plants were treated with viral pathogens or stress-related hormones, such as methyl JA or SA, or caterpillar herbivory (Stotz et al., 2000; Dufresne et al., 2008; Weech et al., 2008). In the current study, *AtACT* was stably expressed within a genotype and not affected by treatment [+/+: *F*_(2,9)_ = 0.26, *p* = 0.77; *pad2-1*: *F*_(2,9)_ = 1.10, *p* = 0.37; *tga2/5/6*: *F*_(2,7)_ = 0.42, *p* = 0.68; Brunner et al., 2004].

### STATISTICAL ANALYSIS

For the redox experiment (repeated independently three times, *n* = 5–10), statistical differences (p í 0.05) in metabolite levels were determined using a two-way analysis of variance (ANOVA) using SPSS version 20 (SPSS Inc.). If a significant time × treatment factor was observed, a one-way ANOVA followed by a Tukey HSD (honestly significant difference) *post hoc* test was conducted to identify the significant difference. The gene expression experiment was repeated twice with two independent biological samples analyzed at each time (total *n* = 3–4). Within each genotype, transcript expression was analyzed by a one-way ANOVA. Statistical differences (p < 0.05) were determined using a Tukey HSD *post hoc* test (Rieu and Powers, 2009). Alternatively, because of the variation inherent with insect feeding studies, a greater than five-fold change in gene expression with respect to control plants was also considered significantly different. Results from statistical analyses are shown in Table A2 in Appendix.

## RESULTS AND DISCUSSION

### ASCORBATE–GLUTATHIONE CYCLE

**FIGURE 1:**
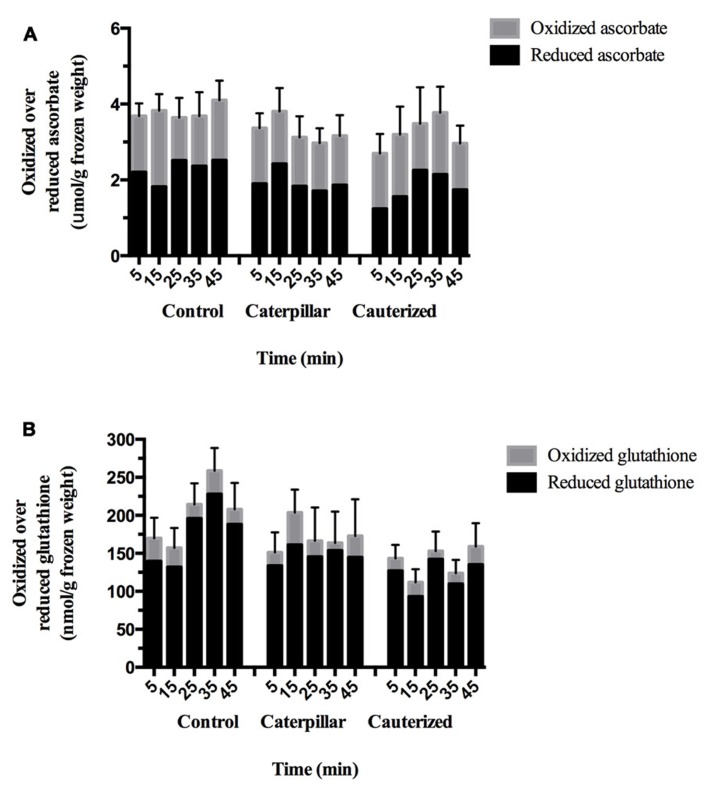
**Time course of redox metabolites in *Arabidopsis* plants subject to caterpillar herbivory.** **(A)** Ascorbate levels. Foliar ascorbate levels in* Arabidopsis* plants subject to herbivory by caterpillars with normal (caterpillar) or impaired salivary secretions (cauterized) compared to control plants. Solid bars represent reduced ascorbate (ASc) levels. Open bars represent oxidized ascorbate (dehydroascorbate, DHA) levels. Values are given in μmol/g frozen weight (FW) and represent means ± SE of three to four independent biological replications. Significant differences in ascorbate were not observed in response to caterpillar herbivory. **(B)** Glutathione levels. Foliar glutathione levels in *Arabidopsis* plants subject to herbivory by caterpillars with normal (caterpillar) or impaired salivary secretions (cauterized) compared to control plants. Solid bars represent reduced glutathione (GSH) levels. Open bars represent oxidized glutathione (GSSG) levels. Values are given in nmol/g FW and represent means ± SE of three to four independent biological replications. Significant differences were determined by two-way ANOVA (**Table A2** in Appendix). At 35 min post-herbivory, a significant reduction in GSSG levels are observed in plants infested by caterpillars, both with normal or impaired salivary secretions, compared to controls. Total and reduced glutathione levels are significantly reduced in caterpillar with impaired salivary secretions compared to control levels.

The ascorbate–glutathione cycle is critical to enable the plant to maintain cellular redox status during stresses, such as insect herbivory (Noctor et al., 2012). Oxidative stress, such as increased H_2_O_2_ levels, may result in either an increase in the levels of total glutathione (glutathione pool) or increased levels of GSSG relative to GSH (redox balance; Noctor et al., 2012). Total ascorbate levels were within the reported physiological range and did not change over the 45 min time course and was independent of treatment (**Figure 1A**; Table A2 in Appendix; Queval and Noctor, 2007). Oxidized and reduced ascorbate levels and the ratio of oxidized ascorbate (DHA)/reduced ascorbate (ASc) did not change in response to caterpillar herbivory. Total glutathione levels were within the expected physiological range and affected by treatment (**Figure 1B**; **Table A2** in Appendix; Queval and Noctor, 2007). Caterpillar herbivory did not affect the oxidized GSSG/reduced GSH ratio but total glutathione levels are lower in plants infested with caterpillars with impaired salivary secretions compared to the control. This likely reflects the reduced glutathione levels found in this treatment. Caterpillar herbivory also had significantly lower oxidized GSSG levels at 35 min post-herbivory; this effect was not salivary-dependent.

Cellular glutathione–ascorbate metabolites levels and/or redox balance are involved in plant defense against pathogens or herbivores (Mou et al., 2003; Ball et al., 2004; Parisy et al., 2007; Schlaeppi et al., 2008; Wünsche et al., 2011; Espunya et al., 2012). The majority of experiments investigating changes in redox metabolites in response to stress (wound, herbivory, pathogens) characterize long-term changes in the cellular oxidative status (Fodor et al., 1997; Mou et al., 2003; Ball et al., 2004; Mateo et al., 2006; Schlaeppi et al., 2008; Gfeller et al., 2011). In this study, we are interested in early changes in cellular antioxidant levels or redox balance (ratio) to caterpillar herbivory that may lead to changes in gene expression. The difficulty in this short-term experiment is to synchronize the initiation and intensity of insect herbivory. Lou and Baldwin (2006) and this study monitored redox metabolites within the first 45 min after the initiation or simulation of herbivory. Lou and Baldwin (2006) noted an increase in H_2_O_2_ levels 30 min after wounding and application of *Manduca sexta* caterpillar regurgitant on *Nicotiana attenuata* leaves. In response to biotrophic pathogens, an increase in total or reduced glutathione levels leads to reduction and activation of NPR1 (Fodor et al., 1997; Mou et al., 2003; Fobert and Després, 2005; Mateo et al., 2006); even though SA injection into leaves shows a transient oxidation of the glutathione pool. In comparison, after wounding, the GSSG/total glutathione ratio increased leading to an activation of the JA pathway (Mhamdi et al., 2010; Gfeller et al., 2011).

Cellular redox changes occur in response to mechanical damage during insect feeding. However, noctuid caterpillar LS, that has been implicated as a stratagem to delay the induction of plant defenses, contains numerous enzymes that may affect cellular redox balance, most notably the H_2_O_2_-producing enzyme GOX (Musser et al., 2002; Weech et al., 2008; Eichenseer et al., 2010). Compared to controls, herbivory by caterpillars with intact salivary secretions did not affect cellular redox balance except for a transient decrease in oxidized GSSG at 35 min (**Figure 1B**). In comparison, reduced glutathione levels were lower in leaves subject to herbivory by caterpillars with impaired salivary secretion compared to controls, indicating oxidative stress. This suggests that the production of H_2_O_2_ by enzymes in the caterpillar LS may act to maintain cellular GSH levels so glutathione does not act further as a signaling molecule (Szalai et al., 2009).

### TRANSCRIPT EXPRESSION IN RESPONSE TO CATERPILLAR HERBIVORY

To explore the link between cellular redox balance and plant responses to caterpillar LS, expression of JA-, ET-, and SA-dependent gene markers were analyzed in wildtype, *pad2-1* mutants, that contain only about 20% of normal glutathione levels, and the *tga2/5/6* triple mutant (Zhang et al., 2003; Parisy et al., 2007). Together with NPR1, TGA transcription factors are activated by a change in redox balance and responsible for SA-dependent gene expression (Després et al., 2003; Mou et al., 2003; Lindermayr et al., 2010). It must, however, be noted that the TGA transcription factors have also been shown to regulate a subset of oxylipin-dependent defensive gene expression (Mueller et al., 2008; Zander et al., 2010).

Jasmonate, SA, and ET play central roles in mediating the plant’s response to caterpillar herbivory (**Figure 3**; Weech et al., 2008; Diezel et al., 2009; Onkokesung et al., 2010). Pré et al. (2008) recently suggested that the transcription factors ORA59 and ERF1 act in parallel pathways to integrate these JA/ET responses. How caterpillar LS manages to manipulate these JA/ET pathways is unknown, but Weech et al. (2008) proposed that caterpillar LS requires an active SA/NPR1 pathway for this strategem. To further complicate issues, recent evidence suggests that ET potentiates SA antagonism with JA and renders it NPR1-independent (Leon-Reyes et al., 2009).

**FIGURE 2:**
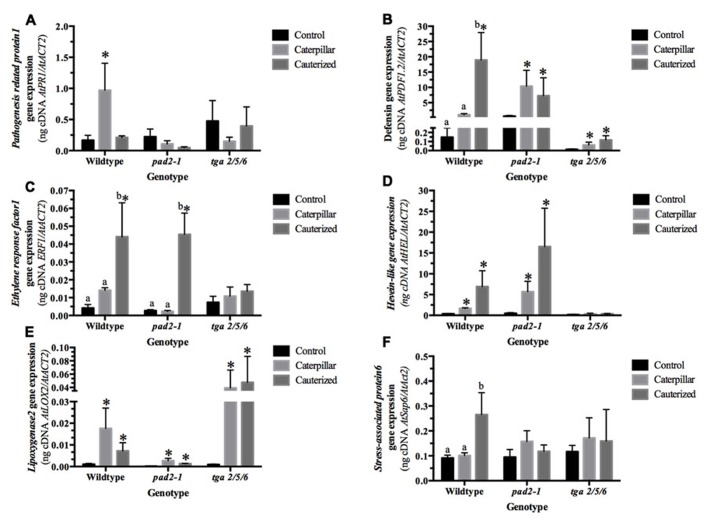
***Arabidopsis* transcript expression in response to caterpillar herbivory analyzed by quantitative real-time reverse transcription PCR.** Seven-week-old *Arabidopsis* plants (Col-0, +/+), *pad2-1* mutant (*pad2-1*), and *tga2/5/6* triple mutant (*tga*) were untreated (control) or subject to herbivory by caterpillars with normal (intact) or impaired (cauterized) salivary secretions for 36 h. From cDNA generated from total RNA, gene-specific primers were used to determine expression levels of **(A)** *AtPR1*, **(B)** *AtPDF1.2*, **(C)** *ERF1*, **(D)** *AtHEL*, **(E)** *AtLOX2*, **(F)** *AtSAP6*. Bars represent the mean values of three to four independent replicates normalized with the reference gene *AtACT2* ± SE. Within each genotype, lower case alphabetical letters indicate significant differences identified by ANOVA followed by a Tukey HSD (p ≤ 0.05; n = 3–4; **Table A2** in Appendix). An asterisk denotes a fivefold or higher change in expression from control levels.

*Pathogenesis-related 1* (*AtPR1*, At2g14610) is a SA-responsive, NPR1-dependent gene marker induced in response to biotrophic pathogen attack and aphid feeding (Glazebrook, 2005; Mur et al., 2006; Kusnierczyk et al., 2007; Walling, 2008; Zhang et al., 1999). In our study, *AtPR1* gene expression was greater than fivefold higher in plants infested by caterpillars with intact LS secretions compared to caterpillars with cauterized spinnerets and control plants, indicating that caterpillar LS secretions result in the activation of SA/NPR1-dependent gene expression (**Figure 2A**; **Table A2** in Appendix). Through activation of the SA pathway by effectors in their LS secretions, *S. exigua* caterpillars are believed to impair the plant’s ability to fully mount a JA-dependent defense response (Weech et al., 2008). Mewis et al. (2006) also observed *AtPR1* expression in *Arabidopsis* response to herbivory by caterpillars of *P. rapae* and *S. exigua*; both these caterpillar LS glands contain redox enzymes, such as GOX (Eichenseer et al., 2010). The increase in *AtPR1* expression was alleviated in *pad2-1* and *tga2/5/6* mutant plants, in line with previous studies showing that glutathione and the TGA transcription factors are upstream signals in *AtPR1* expression (Després et al., 2003; Mou et al., 2003; Lindermayr et al., 2010).

Expression of the gene encoding plant defensin, *AtPDF1.2b* (At2g26020), is induced by treatment of plants with JA and ET working synergistically through ORA59 (Penninckx et al., 1998; Pré et al., 2008); however, antagonism between MYC2 and ERF1 regulation of *AtPDF1.2* is proposed to reflect MYC2 regulation of *ERF1* expression (Dombrecht et al., 2007). As well, SA-dependent suppression of *AtPDF1.2* expression requires active NPR1 and TGA transcription factors (Spoel et al., 2003; Ndamukong et al., 2007; Koornneef et al., 2008). ET modulates this SA–JA antagonism; NPR1-dependent antagonism of the expression of JA-dependent genes, such as *AtPDF1.2*, becomes NPR1-independent in the presence of ET (Leon-Reyes et al., 2009).

In wildtype plants, an 18-fold increase in *AtPDF1.2* transcript expression is observed in response to herbivory by caterpillars with impaired salivary secretions compared to normal caterpillars or control plants, in agreement with previous studies that caterpillar LS suppresses JA-dependent plant defenses (**Figure 2B**; **Table A2** in Appendix; Musser et al., 2002; Weech et al., 2008). In *pad2-1* and *tga2/5/6* mutants, LS-mediated restraint of *AtPDF1.2* expression is not observed, indicating that glutathione and TGA transcription factors are required for the suppression of plant induced defenses by caterpillar herbivory. In *pad2-1* mutants, a 12.5-fold increase in *AtPDF1.2* levels is seen in plants infested by caterpillars compared to controls. The lower glutathione levels in the *pad2-1* mutant may impair the activation of a pathway, such as the reduction of NPR1 and/or TGA transcription factors, which are needed for the LS-mediated suppression of plant defenses (Mou et al., 2003; Fobert and Després, 2005). A fivefold increase in *AtPDF1.2* expression is seen in plants fed upon by caterpillars compared to controls in the *tga2/5/6* mutant plants. However, it must be noted that TGA transcription factors also regulate the late expression (~48 h) of a subset of JA-dependent genes, such as *AtPDF1.2* (Zander et al., 2010). Perhaps, a strong difference in gene expression between normal and cauterized caterpillars is not observed because of the requirement for TGA transcription factors, although a fivefold increase in expression is observed in caterpillar-infested *tga2/5/6* mutants compared to controls. These results suggest that caterpillar LS-dependent suppression of JA-mediated activation of *AtPDF1.2* gene expression is dependent on glutathione levels and, perhaps, the activation of TGA transcription factors.

In wildtype plants, results correlate with previous observations that glutathione negatively regulates *AtPDF1.2* expression (Koornneef et al., 2008); we also observed that wildtype plants infested by caterpillars with impaired salivary secretions had lower reduced glutathione compared to controls and, consequently, higher *AtPDF1.2* expression (**Figures 1B2B**). Also, the LS-associated negative regulation of *AtPDF1.2* is alleviated in the *pad2-1* mutant. Our observation that this LS-mediated suppression of *AtPDF1.2* is lessened in the *tga2/5/6* triple mutant supports observations that suppression of *AtPDF1.2* gene expression requires the interaction of glutaredoxin480 with TGA transcription factors (Ndamukong et al., 2007; Koornneef and Pieterse, 2008; Zander et al., 2011). ET also plays a role in modulating the mechanism of SA/NPR1 inhibition of JA-dependent responses (Leon-Reyes et al., 2009); in the presence of ET, this suppression becomes NPR1-independent. However, given the links to glutathione and, possibly TGA transcription factors, and previous research, our data points to a LS-mediated NPR1-dependent inhibition of *AtPDF1.2* gene expression (Weech et al., 2008).

Alternatively, current models propose that JA-dependent inhibition of *AtPDF1.2* expression may be mediated through the negative regulation of *ERF1* (At3g23240) by MYC2 (Dombrecht et al., 2007; (Zander et al., 2010). Therefore, *ERF1 *expression was measured to determine if it was mirrored by *AtPDF1.2* expression. As seen with *AtPDF1.2*, a significant increase in *Arabidopsis* *ERF1* transcript expression is observed in response to herbivory by caterpillars with impaired LS secretions compared to normal caterpillars or control plants (**Figures 2B, C**; **Table A2** in Appendix); however, this LS-mediated suppression of ERF1 is also observed in the *pad2-1* mutants. The distinct patterns between *AtPDF1.2* and *ERF1* expression suggest LS-mediated regulation is likely not reflective of MYC2 antagonism of *ERF1*; however, they suggest that there may be LS-linked, an ET, glutathione-independent mechanism of suppression. LS-suppression of *ERF1* is alleviated in the *tga2/5/6* triple mutant. Zander et al. (2010) found that TGA transcription factors may suppress *ERF1* expression.

*Hevein-like* (*AtHEL*, PR4, At304720) gene expression is a marker of the ORA59 branch of the JA/ET signaling pathways (Potter et al., 1993; Dombrecht et al., 2007; Pré et al., 2008; Verhage et al., 2011; Zarei et al., 2011). In comparison to *AtPDF1.2*, suppression of JA-linked *AtHEL* expression by the SA pathway is NPR1-independent (Ndamukong et al., 2007). In wildtype and *pad2-1* mutant plants, over a fivefold increase in gene expression is observed in plants infested by caterpillars compared with controls (**Figure 2D**); however, a LS effect is not observed (**Table A2** in Appendix). These results support the argument that caterpillar LS-mediated suppression of induced plant defenses is glutathione- and NPR1-dependent. Unexpectedly, this caterpillar-mediated *AtHEL* expression was at basal levels in the *tga2/5/6* triple mutant plants, suggesting that these transcription factors may be involved in regulation of *AtHEL* expression.

The gene encoding *lipoxygenase2* (*AtLOX2*, At3g45410) is an early expression marker of the JA-responsive MYC2 branch (Bell et al., 1995; Dombrecht et al., 2007). As has been observed previously, *AtLOX2* levels are induced sevenfold in response to insect herbivory and a LS gland-specific difference in gene expression is not observed (**Figure 2E**; **Table A2** in Appendix; Weech et al., 2008). This same pattern was observed in *pad2-1* and *tga2/5/6* mutant plants. Though regulated by MYC2, the strong upregulation of this early gene occurs before SA/NPR1-mediated cross-talk (Mou et al., 2003; Spoel et al., 2003; Ndamukong et al., 2007; Koornneef and Pieterse, 2008; Spoel and Dong, 2008; Tada et al., 2008; Leon-Reyes et al., 2010). As well, LS-associated post-transcriptional modifications of LOX2 may regulate activity rather than gene expression (Thivierge et al., 2010).

The stress-associated *AtSAP6* (At3g52800) was induced in plants fed upon by caterpillars with impaired LS secretions compared to controls (**Figure 2F**; **Table A2** in Appendix). This difference was alleviated in the *pad2-1* and the *tga2/5/6* triple mutants indicating the possible involvement of glutathione and TGA transcription factors in the regulation of expression of this gene. *AtSAP6* is strongly induced in response to numerous stresses, such as wounding and herbivory by caterpillars of the specialist *P. rapae* (Reymond et al., 2004; Ströher et al., 2009); however, in response to herbivory, this transcript was induced in both the wildtype and the JA-perception impaired *coi1-1 gl1* mutant implying that JA signaling is not required for the expression of this gene.

## CONCLUSION

**FIGURE 3:**
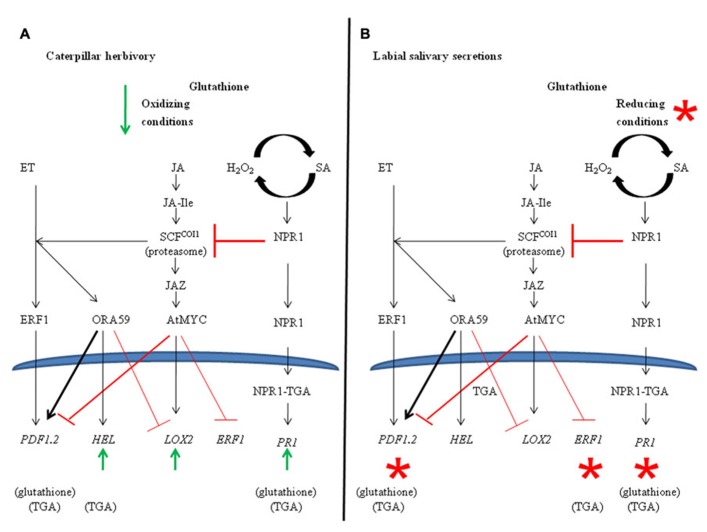
**Model of ethylene-, jasmonate-, and salicylate-dependent pathway illustrating major cross-talk signaling nodes and marker genes.** **(A)** Changes in redox metabolites and gene expression in response to caterpillar herbivory. Illustrates caterpillar herbivory-dependent changes compared to control plants. Cellular GSSG, which is linked to the induction of JA-defenses, decrease transiently 35 min after caterpillar herbivory. Markers of the SA, ORA59, and AtMYC2 pathway, respectively *AtPR1*, *AtHEL*, and *AtLOX2*, are induced in response to herbivory. **(B)** Proposed model caterpillar labial saliva (LS) mediated suppression of jasmonate-dependent responses. Illustrates LS-associated changes between plants fed on by caterpillars with intact vs. impaired salivary secretions. Asterisks indicate LS-associated changes. Herbivory by caterpillars with impaired salivary secretions result in cellular oxidative stress (lower total and reduced cellular GSH levels) compared to controls. Induction of the *AtPR1* and suppression of *AtPDF1.2 *and *ERF1* are LS-dependent. Involvement of glutathione orTGA transcription factors are indicated in brackets underneath the marker gene.

Plant responses to insect herbivory are mediated through carefully regulated, complex hormone-mediated interactions. Herbivory by *S. exigua* caterpillars attenuate these JA-dependent plant defense responses; a mechanism believed to be related to LS-associated secretions (Musser et al., 2002; Weech et al., 2008). Given the presence of GOX in the LS of this caterpillar, the relationship between LS secretions and changes in cellular redox potential was investigated. Changes in cellular oxidative stress and, in particular, the GSSG/total glutathione ratio are signals for the induction of JA-dependent defenses (Szalai et al., 2009; Gfeller et al., 2011). Herbivory by caterpillars with intact salivary secretions did not affect cellular redox balance, except for a transient decrease in oxidized GSSG at 35 min (**Figure 3**). In comparison, herbivory by caterpillars with impaired salivary secretions resulted in an increase in cellular oxidative status through a decrease in reduced glutathione levels. In support of this, genes, such as *AtPR1* and *AtPDF1.2*, showed LS-dependent transcript expression that was alleviated in the *pad2-1 *and *tga2/5/6* triple mutant (**Figures 2A, B and 3**).

Increased expression of *AtPR1* by herbivory using caterpillars with intact salivary secretions support the notion that LS-mediated attenuation of JA responses acts through cross-talk with the SA/NPR1 pathway (**Figure 3**). As well, even though *AtPDF1.2* is a JA/ET marker, recent studies have shown that mid- to late-gene expression is regulated by TGA transcription factors (Zander et al., 2011). Therefore, suppression of *AtPDF1.2* gene expression by caterpillar LS may also reflect cross-talk between JA- and SA/NPR1 pathways. The LS-associated modulation of ET-dependent genes, *ERF1* and *AtHEL*, show disparate regulation since *ERF1* expression shows glutathione-independent, LS-associated suppression whereas a LS-dependent difference in *AtHEL* is not observed (**Figures 2C, D**). This may support recent evidence that the ET pathway is mediated through two distinct branches regulated by ORA59 or ERF1 transcription factors (Pré et al., 2008). In fact, *Pieris rapae* caterpillar oral secretions, which are a mixture of gut-derived regurgitant, secretions from the ventral eversible gland and salivary secretions from the mandibular and labial glands, specifically activate the ORA59 branch of the JA/ET pathway leading to the suppression of MYC2-dependent defenses (Felton, 2008; Hogenhout and Bos, 2011; Verhage et al., 2011; Zebelo and Maffei, 2012). These caterpillars also show a feeding preference for plants that overexpress ORA59.

The mechanism behind this LS-mediated cross-talk may be explained by the model recently proposed by Van der Does et al. (2013). In an elegant set of experiments, these authors systematically demonstrated that suppression of the JA-induced pathway by the SA/NPR1 pathway occurs downstream of SCF^COI1^-mediated protein degradation. Instead, the SA/NPR1 pathway negatively regulates the expression of *AtPDF1.2* by affecting the accumulation of the ORA59 transcription factor. Our data from this and previous studies also show that caterpillar LS-mediated suppression of *AtPDF1.2* is SA/NPR1 pathway mediated and does not appear to involve cross-talk between the ERF1 pathway (Weech et al., 2008); therefore, further studies investigating ORA59 protein levels in this plant–insect system needs to be investigated.

## Conflict of Interest Statement

The authors declare that the research was conducted in the absence of any commercial or financial relationships that could be construed as a potential conflict of interest.

## APPENDIX

**Table A1:** Quantitative real-time polymerase chain reaction primers.

**Gene**	**Accession number**	**Annealing temperature (°C)**	**Forward (5′–3′)**	**Reverse (5′–3′)**	**Reference**
*ERF1*	At3g23240	62	GAC GGA GAA TGA CCA ATA AGA AG	CCC AAA TCC TCA AAG ACA ACT AC	Swarup et al. (2007)
*AtHEL*	At304720	57	CAA GTG TTT AAG GGT GAA GA	CGG TGT CTA TTT GAT TGA AC	Conn et al. (2008)
*AtLOX2*	At3g45410	57	GTC CTA CTT GCC TTC CCA AAC	ATT GTC AGG GTC ACC AAC ATC	Weech et al. (2008)
*AtPDF1.2b*	At2g26020	59	CGG CAA TGG TGG AAG CA	CAT GCA TTA CTG TTT CCG CAA	Jirage et al. (2001)
*AtPR1*	At2g14610	62	CAC TAC ACT CAA GTT GTT TGG A	CAT GCA TTA CTG TTT CCG CAA A	Primer3
*AtSAP6*	At3g52800	63	TCA ACG CAT CGA ACG GCT CTG A	GCG AAA GCG AAT CCG TTG GTG AAA	Primer3
*AtACT2*	At3g18780		ACC AGC TCT TCC ATC GAG AA	GAA CCA CCG ATC CAG ACA CT	Dufresne et al. (2008)

**Table A2:** Statistical results of plant–insect experiments.

**Ascorbate**		
Total	Range: 2.69–3.98 μmol/g FW	Effect of treatment, *F*(_2,103_) = 1.33, p = 0.27; effect of time, *F*(_4,103_) = 0.16, p = 0.96; interaction, *F*(_8,103_) = 0.66, p = 0.73
Oxidized (DHA)	Range: 1.22–1.83 μmol/g FW	Effect of treatment, *F*(_2,100_) = 0.41, p = 0.66; effect of time, *F*(_4,100_) = 0.90, p = 0.47; interaction, *F*(_8,100_) = 0.43, p = 0.90
Reduced (Asc)	Range: 1.23–2.81 μmol/g FW	Effect of treatment, *F*(_2,105_) = 2.50, p = 0.09; effect of time, *F*(_4,105_) = 0.58, p = 0.68; interaction, *F*(_8,105_) = 0.97, p = 0.47
Oxidized/reduced (DHA/Asc)		Effect of treatment, *F*(_2,100_) = 1.62, p = 0.21; effect of time, *F*(_4,100_) = 0.1.30, p = 0.28; interaction, *F*(_8,100_) = 1.11, p = 0.36
**Glutathione**
Total	Range: 151–226 nmol/g FW	Effect of treatment, *F*(_2,109_) = 3.35, p = 0.04; effect of time, *F*(_4,109_) = 0.32, p = 0.86; interaction, *F*(_8,109_) = 0.16, p = 0.99; **Figure 2A**)
Oxidized (GSSG)	Range: 9.9–42.6 nmol/g FW	Effect of treatment, *F*(_2,89_) = 3.31, p = 0.04; effect of time, *F*(_4,89_) = 2.10, p = 0.09; interaction, *F*(_8,89_) = 2.24, p = 0.03. Since interaction was significant, this was followed by a one-way ANOVA to determine the time point where there was a significant difference: 5 min: *F*(_2,19_) = 2.69, p = 0.09; 15 min: *F*(_2,14_) = 1.91, p = 0.18; 25 min: *F*(_2,19_) = 2.64, p = 0.10; 35 min: *F*(_2,20_) = 3.76, p = 0.04; 45 min: *F*(_2,19_) = 0.53, p = 0.60
Reduced (GSH)	Range: 93.2–227.9 nmol/g FW	Effect of treatment, *F*(_2,87_) = 3.42, p = 0.04; effect of time, *F*(_4,87_) = 0.67, p = 0.62; interaction, *F*(_8,87_) = 0.56, p = 0.81
Oxidized/reduced (GSSG/GSH)		Effect of treatment, *F*(_2,87_) = 0.99, p = 0.37; effect of time, *F*(_4,87_) = 2.18, p = 0.08; interaction, *F*(_8,87_) = 1.07, p = 0.39
**Gene expression**	**Genotype**
*AtPR1*	Wildtype	*F*_(2,8)_ = 4.44, p = 0.05; fivefold increase in gene expression in plants attacked by caterpillars with intact labial salivary secretions compared to control plants or between control plants or plants infested by caterpillars with impaired salivary secretions
	*pad2-1*	*F*_(2,9)_ = 1.23, p = 0.32
	*tga2/5/6*	*F*_(2,7)_ = 0.35, p = 0.72
*AtPDF1.2*	Wildtype	*F*_(2,8)_ = 6.00, p = 0.03; 18-fold increase in gene expression in response to herbivory by caterpillars with impaired salivary secretions compared to normal caterpillars or controls
	*pad2-1*	*F*_(2,8)_ = 1.50, p = 0.28; 12.5-fold increase in gene expression is seen between plants infested by caterpillars compared to controls
	*tga2/5/6*	*F*_(2,7)_ = 3.31, p = 0.10; fivefold increase in gene expression is seen in plants fed upon by caterpillars compared to controls
*ERF1*	Wildtype	*F*_(2,8)_ = 5.07, p = 0.04; 10-fold increase in gene expression seen in plants fed upon by caterpillars with impaired salivary secretions compared to control plants
	*pad2-1*	*F*_(2,9)_ = 12.83, p = 0.002; 20-fold increase in gene expression seen in plants fed upon by caterpillars with impaired salivary secretions compared to control plants or plants attacked by caterpillars with labial salivary secretions
	*tga2/5/6*	*F*_(2,7)_ = 0.61, p = 0.57
*AtHEL*	Wildtype	*F*_(2,9)_ = 2.50, p = 0.14; fivefold increase in gene expression is observed in plants infested by caterpillars compared with control
	*pad2-1*	*F*_(2,9)_ = 2.22, p = 0.17; 10-fold increase in gene expression is observed in plants infested by caterpillars compared with controls
	*tga2/5/6*	*F*_(2,7)_ = 3.31, p = 0.10
*AtLOX2*	Wildtype	*F*_(2,7)_ = 1.48, p = 0.29; sevenfold increase in gene expression is observed in plants infested by caterpillars compared with controls
	*pad2-1*	*F*_(2,9)_ = 3.68, p = 0.07; sevenfold increase in gene expression is observed in plants infested by caterpillars compared with controls
	*tga2/5/6*	*F*_(2,7)_ = 1.16, p = 0.37; 40-fold increase in gene expression is observed in plants infested by caterpillars compared with controls
*AtSAP6*	Wildtype	*F*_(2,8)_ = 5.02, p = 0.04
	*pad2-1*	*F*_(2,9)_ = 0.85, p = 0.46
	*tga2/5/6*	*F*_(2,7)_ = 0.14, p = 0.87

*A two-way ANOVA followed by a Tukey HSD was used to evaluate redox metabolite levels over a 45-min time course. A one-way ANOVA was used to evaluate differences in gene expression within each genotype. A fivefold or higher difference in gene expression is also indicated.*

## Abbreviations

1D: one-dimensional
2D: two-dimensional
2,4-D: 2,4-dichloroacetic acid
2,4-DB: 2,4-dichlorobutyric acid
ACX: acyl-CoA oxidase
BA: benzoic acid
BSA: bovine serum albumin
CDS: coding sequence
co-IP: co-immunoprecipitation
DRP: dynamin-related protein
ER: endoplasmic reticulum
ERPIC: ER/peroxisome intermediate compartment
ESI: electrospray ionization
EST: expressed sequence tag
FFE: free-flow electrophoresis
FIS: fertilization-independent seed
GFP: green fluorescent protein
GPK: glyoxysomal protein kinase
IAA: indole-3-acetic acid
IBA: indole-3-butyric acid
ICAT: isotope coded affinity tags
ICL: isocitrate lyase
IDHP: NADP-dependent isocitrate dehydrogenase
IEF: iso-electric focusing
iTRAQ: isobaric tags for relative and absolute quantitation
LACS: long chain acyl-CoA synthetase
MALDI: matrix-assisted laser desorption/ionization
MFP: multi-functional protein
MRM: multiple reaction monitoring
MS: mass spectrometry
MVA: mevalonic acid
NADK: NAD kinase
PAGE: polyacrylamide gel electrophoresis
PMF: peptide mass fingerprint
PTS: peroxisome targeting signal
RNAi: ribonucleic acid interference
RNS: reactive nitrogen species
ROS: reactive oxygen species
SA: salicylic acid
SDS: sodium dodecyl sulfate
SILAC: stable isotope labeling by amino acids in cell culture
SRM: selected reaction monitoring
TAP: tandem affinity purification
TMT: tandem mass tags
